# Diagnosis and treatment of HS after endoscopic thyroid surgery: case report and brief literature review

**DOI:** 10.3389/fsurg.2023.1267701

**Published:** 2023-09-28

**Authors:** Yuqing Deng, Guoqian Ding

**Affiliations:** Department of General Surgery, Beijing Friendship Hospital, Capital Medical University, Beijing, China

**Keywords:** Horner’s syndrome, thyroid neoplasms, postoperative complications, diagnosis, case report

## Abstract

**Background:**

Horner’s syndrome (HS) is a rare condition due to damage to the 3-neuron sympathetic pathway anywhere between the posterior-lateral nuclei of the hypothalamus and the oculosympathetic fiber, particularly as a post-thyroidectomy symptom. In this case report, we present a case of HS following endoscopic thyroid surgery (ETS) and briefly review the literature.

**Case report:**

During a routine physical examination, a 29-year-old female patient was incidentally found to have multiple nodules in the right thyroid. She was subsequently admitted to the Department of General Surgery for further examinations and treatment. A fine-needle aspiration biopsy confirmed malignancy in these nodules. As a result, the patient underwent radical resection of the right thyroid and ipsilateral central lymph node dissection using endoscopy. Pathological diagnosis revealed papillary thyroid carcinoma. Unexpectedly, on the third day after the operation, the patient was diagnosed with Horner’s syndrome based on the presence of miosis and ptosis. After 1 week of follow-up, the symptoms related to HS significantly improved.

**Conclusion:**

Horner’s syndrome is an uncommon complication of thyroidectomy in patients undergoing ETS. Therefore, it is crucial to perform careful operations and minimize iatrogenic surgical damage to reduce the incidence of HS. This case serves as a reminder that making rational judgments and implementing appropriate measures are essential for achieving a favorable prognosis and preserving facial esthetics.

## 1. Introduction

Horner’s syndrome (HS) occurs due to disruption of the 3-neuron oculosympathetic nerve pathway (1). It manifests when there is an unexpected interruption in any tier of this pathway. The prominent clinical features include ptosis (drooping eyelid), miosis, and the rare but characteristic symptom of ipsilateral anhidrosis. HS is commonly secondary to compression caused by thyroid masses (2) or iatrogenic injury to the cervical sympathetic nerve pathway during cervical or thoracic surgery (3). More than 20 cases of post-thyroidectomy HS have been reported, with endoscopy-related HS being particularly notable (4). As endoscopic techniques continue to advance, patients increasingly opt for esthetic and minimally invasive treatments. However, it is important to acknowledge that hidden risks may occur during endoscopic procedures.

In this report, we present the case of a female patient who developed HS following endoscopic thyroid dissection. In addition, we discuss the anatomical and etiological mechanisms of postoperative HS, as well as its clinical diagnosis and treatment, based on a thorough review of the available literature and our own previous experiences.

## 2. Case description

**Figure 1:**
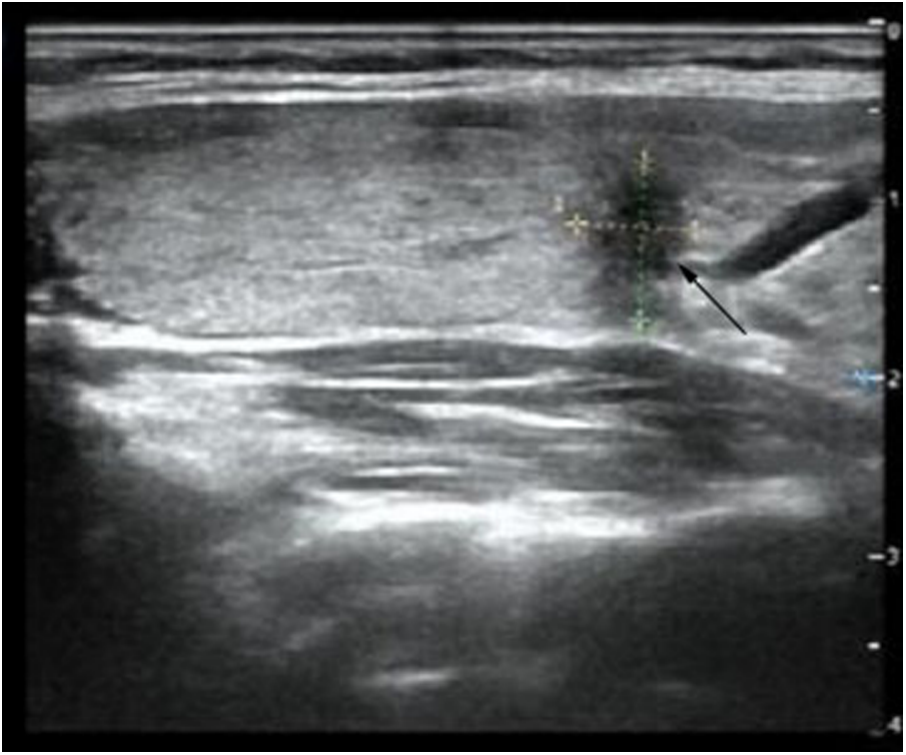
Ultrasound image expresses a thyroid nodule (0.70 cm × 0.70 cm × 0.90 cm) located at the inferior thyroid on the right side (shown by a black arrow).

A 29-year-old female patient was admitted to the Department of General Surgery after discovering multiple thyroid nodules on the right side during a routine medical examination half a month ago (Figure 1). The largest nodule, located at the inferior thyroid, measured 0.70 cm × 0.70 cm × 0.90 cm. The Thyroid Imaging Reporting and Data System (TI-RADS) score classified the largest nodule as 6, while the rest were scored as 3. The patient has no chronic diseases or hereditary cancer-related symptoms and did not report any relevant discomfort such as dyspnea, odynophagia (swallowing pain), cough, or hoarseness during the medical history collection. As part of the diagnostic process, the patient underwent a fine-needle aspiration biopsy of the suspicious nodule to determine its nature. Unfortunately, the results revealed a malignancy.

**Figure 2:**
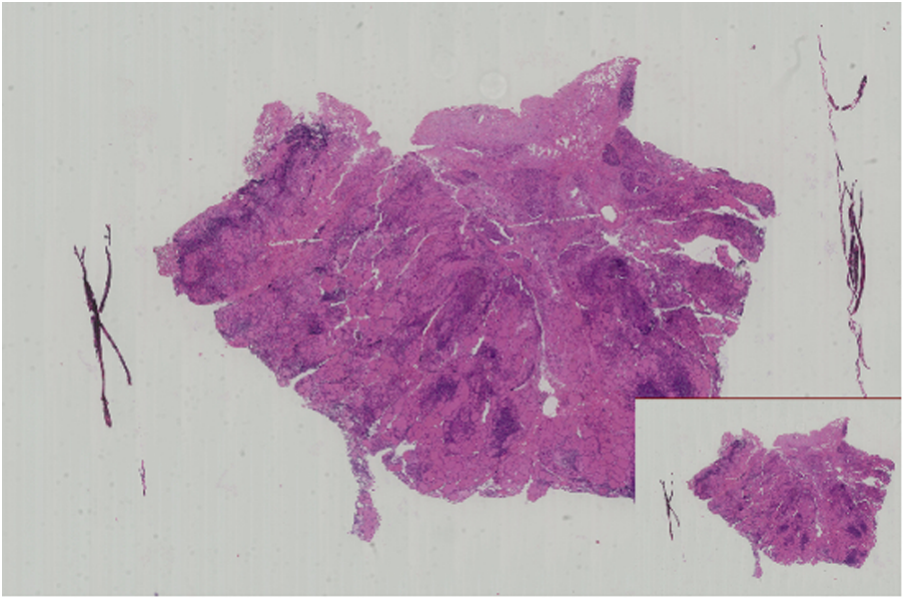
Microscopic image of this patient shows papillary thyroid carcinoma (H&E staining, ×20 magnification).

Considering the significant cosmetic and minor traumatic advantages of microtrauma surgery compared to its negative aspects, the patient decided to undergo endoscopic thyroid surgery (ETS) using a bilateral three-channel endoscopy approach on the third day of hospitalization. After conducting a preoperative evaluation, a right thyroid malignancy (cT1aN0M0) was diagnosed. Following the latest 2022 version of the guidelines for diagnosing and treating thyroid cancer (5), we opted for a surgical approach involving the right thyroid lobe, isthmus resection, and right central lymph node dissection. Before this, we conducted thorough communication with the patient and their family to explain the risks associated with the surgery and the available surgical options. The patient and their family expressed their understanding and provided informed consent by signing the necessary documents. The surgical field was positioned in close proximity to the two nipples, with an incision on the left side and two incisions on the contralateral area of the anterior chest wall. Three trocars were used to establish access channels. Subsequently, the surgeon carefully detached the platysma muscle from the surgical field, working above the level of the thyroid cartilage and laterally toward the middle of the sternocleidomastoid muscle. A coagulation hook was utilized to make a lengthwise incision along the neck white line, followed by dissecting forceps to bluntly separate the anterior cervical muscles and expose the intact right lobe of the thyroid gland. Prior to this, the thyroid isthmus was removed to obtain sufficient visibility of the trachea. While preserving the posterior branches of the thyroid vessels, the inferior thyroid artery and vein branches were interrupted and ligated. Throughout the entire thyroidectomy procedure, great care was taken to avoid any damage to the recurrent laryngeal nerve with the assistance of a nerve monitoring device. Samples were sent for frozen section biopsies (FSBs), including the thyroid isthmus and right-side nodules. The FSB results confirmed the presence of papillary thyroid carcinoma (PTC, Figure 2). Based on this diagnosis, ipsilateral central lymph nodes were subsequently removed. The postoperative pathological examination confirmed the initial FSB findings and identified metastasis in two out of five central lymph nodes.

**Figure 3:**
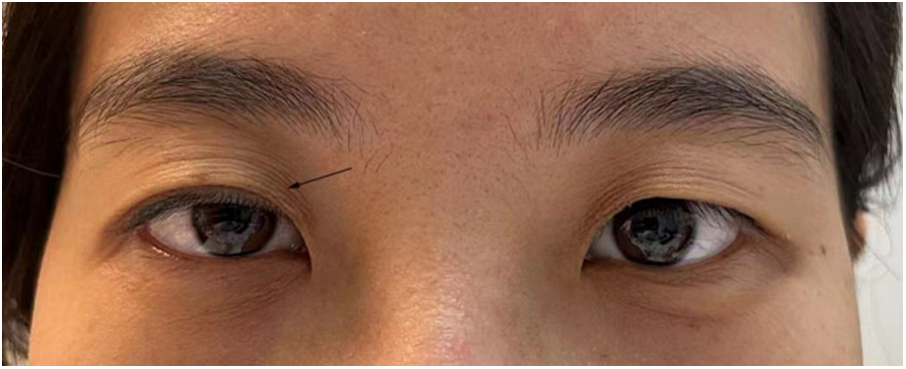
A patient suffered from right-side ptosis after acceptancy of ETS (shown by a black arrow).

On the third day following the surgery, the patient experienced a sudden weakness in the right upper eyelid (Figure 3). In response, a specialized neurologist performed a comprehensive ocular examination. The right pupil displayed noticeable constriction compared to the left pupil (right: 3 mm, left: 4.5 mm). Subtle ipsilateral anhidrosis was observed during the neurological assessment. Based on these findings, a diagnosis of Horner’s syndrome was established.

Consequently, the patient received accurate and cautious neurotropic treatment, which involved the administration of Mecobalamin (0.5 mg, orally, three times daily) along with vitamin B1 (10 mg, orally, once daily) for a continuous period of 5 days.

During the 1-week follow-up, the previous symptoms of HS showed visible improvement without any recurrence or exacerbation. The patient expressed gratitude for the remarkable prognosis, timely notification, and effective treatment received (Table 1).

**Table 1:** The timeline of the diagnosis and treatment.

Timeline	Diagnosis and treatment
June 15, 2023	Physical examination revealed thyroid nodules
June 30, 2023	Fine-needle aspiration identified malignancy
July 2, 2023	Comprehensive department internal discussion
July 3, 2023	Endoscopic thyroidectomy was performed
July 6, 2023	The ptosis and ipsilateral anhidrosis was discovered on the right side
July 6, 2023	HS was diagnosed
July 6, 2023	Neurotropic treatment was performed
July 7, 2023	Discharged from the hospital
July 14, 2023	The symptoms of HS were relieved

## 3. Discussion

### 3.1. Definition and epidemiology of Hs

HS is a rare condition characterized by the classic symptoms of ptosis, miosis, and ipsilateral anhidrosis. The term “Horner’s syndrome” was coined by Swiss ophthalmologist Johann Friedrich Horner in 1869. The prevalence of HS is estimated to be approximately one in 6,000 individuals. However, postoperative HS following thyroidectomy is even more uncommon, with an incidence rate of approximately 0.2% among relevant postoperative complications, as reported in existing research (6).

### 3.2 Endoscopic thyroidectomy approaches

Endoscopic thyroidectomy approaches include the gasless transaxillary endoscopic thyroidectomy (GTET), bilateral axillo-breast approach (BABA), retro-auricular (RA) facelift approach, and transoral endoscopic thyroidectomy via vestibular approach (TOETVA) (7). Laparoscopic surgery has been proven to be comparable to open surgery in terms of therapeutic effectiveness and safety. According to the latest meta-analysis, with the exception of GTET, the operation time for laparoscopic surgery is longer than that for open surgery. However, the two approaches have no significant difference in the overall length of hospital stay and the incidence of postoperative laryngeal nerve damage or hypocalcemia (7, 8). As a minimally invasive procedure, laparoscopic surgery can minimize postoperative neck scars and achieve cosmetic results (9), making it a preferred option for many patients. Surgical energy equipment plays a crucial role in laparoscopic thyroidectomy. This procedure involves operating within a confined surgical field through a small incision. The high-energy thermodynamic surgical machinery generates sufficient energy to quickly cut and separate blood vessels or tissues approximately 7 mm in diameter. In addition, it denatures collagen and elastin while promoting coagulation of blood vessels to effectively control bleeding.

### 3.3. Anatomy and etiology of post-thyroidectomy HS

HS is known to result from an impairment of the oculosympathetic nerve pathway. Understanding the anatomy of this pathway is crucial for comprehending the development of postoperative HS. The oculosympathetic nerve pathway consists of three-order neurons originating from the hypothalamus. The first-order neuron descends along the axon to the lower cervical or upper thoracic spinal cord at approximately the C8, T1, and T2 levels (10). The second-order neuron arises from the spinal cord nucleus and its axon ascends to the superior cervical ganglion near the bifurcation of the common carotid artery. The inferior thyroid artery is in close proximity to the intermediate ganglion (11). Subsequently, ascending fibers, vasomotor fibers, and sweat gland–distributed nerves form the oculosympathetic fiber, enabling them to independently carry out their specific functions (12). Various factors can lead to different levels of Horner’s syndrome based on the involvement of the first-, second-, or third-order neurons. Lesions within the intracranial or skull base region, such as brain tumors, cerebral ischemia, or cerebral hemorrhage, can potentially cause first-order neuron HS. Surgical procedures involving the cervical or thoracic regions can result in HS by affecting the second-order neuron of the cervical sympathetic nerve (13). Orbit diseases that affect the oculosympathetic fiber are associated with third-order neuron HS.

Although the risk factors can be conceptualized based on the anatomical structures involved, precise consequences can only be explained in a fraction of clinical cases. Therefore, further research is needed to fully understand the etiology and intricacies of post-thyroidectomy Horner’s syndrome (14).

With the increasing popularity of ETS, there is a growing demand for procedures that combine esthetic outcomes with radical cure. However, it is important to note that the incidence of postoperative HS following ETS is higher compared to open thyroid surgery. The association between HS and ETS primarily lies in iatrogenic injury during the thyroidectomy procedure. The consequences of such injuries must be considered based on the location and characteristics of the nerves involved in ETS (13). First, in endoscopic surgery, the surgical field is limited, making obtaining a comprehensive view of the complete branches of vessels and nerves within a narrow visual field challenging. Furthermore, retractors used to expose the thyroid gland can inadvertently cause direct, blunt damage to adjacent tissues or organs, including the carotid artery and sympathetic nerves surrounding the thyroid gland. In certain individuals, oculosympathetic branches may adhere to the thyroid gland, and there may also be communication branches between cervical nerves and recurrent laryngeal nerves, increasing the likelihood of unintentional injury to the oculosympathetic fiber. Ligating the inferior thyroid artery also poses a risk of damaging adjacent sympathetic nerves (4). Another contributing factor is the potential thermal injury caused by thermodynamic devices. Advanced surgical instruments such as ultrasonic knives and bipolar electrocautery provide convenient and precise operations. However, endoscopy relies more on heat conduction compared to conventional surgery. The cervical sympathetic nerve is particularly vulnerable to heat-related damage.

To minimize the risk of postoperative HS in patients with ETS, surgical techniques should be employed with meticulous precision and care to protect the delicate nerves and blood vessels in the surgical field. Further research and improvements in surgical approaches are necessary to reduce the occurrence of HS and other complications associated with ETS.

It is important to note that the closure of the carotid artery sheath with an ultrasonic knife for separating the thyroid gland from the surrounding lymphoid and adipose tissue may result in temporary or permanent nerve damage due to heat conduction (15). In addition, there are other factors that can potentially cause Horner’s syndrome, including but not limited to thyroid neoplasms or huge goiter compression (3), arteritis (16), central nervous system infection (3), peridural obstetric anesthesia (17, 18), and occasionally lung transplantation.

In conclusion, it is crucial to be aware of the potential risks of Horner’s syndrome during endoscopic thyroid surgery.

### 3.4 Clinical appearances

As mentioned earlier, HS is closely related to dysfunction of the sympathetic nervous system. It primarily manifests externally through oculosympathetic lesions at specific anatomical sites. The classical symptoms of HS include upper eyelid ptosis, miosis, and ipsilateral facial or chest wall anhidrosis, which can occur together or separately and serve as important indicators for clinical diagnosis. Organs located near the damaged nerves’ distribution areas are susceptible to impairment, and the severity of the impairment depends on the extent of damage to the sympathetic nerves. For example, the motion of pupil constriction is mainly regulated by parasympathetic nerves, while the activation of the sympathetic nerves controls dilation. Consequently, miosis, which results from parasympathetic function, prevails in 3-neuron oculosympathetic lesions. In addition to the example mentioned above, the activity of periocular muscles corresponds to the neural network distributed in the orbit. Activating the levator palpebrae superior muscle allows the upper eyelid to maintain a raised position. Therefore, if there is damage to the sympathetic nerves, the eyelid may droop, resulting in noticeably unequal palpebral fissure size. Another symptom of HS is anhidrosis; although uncommon, it is considered a classical clinical manifestation. The sympathetic system controls facial sweating, so perspiration dysfunction is a facial sign that aids diagnosis.

It is important to note that postoperative HS is usually reversible and not a significant obstacle. However, the long-term prognosis remains uncertain and requires careful monitoring during follow-up (19).

### 3.5 Diagnosis and treatment of HS

First and foremost, the patient's complaint raises an undeniable alarm, highlighting the necessity for a thorough diagnosis. It is crucial to consider both the present and past medical history to distinguish whether the condition is primary or secondary. The etiology of the condition requires careful investigation and collaboration with prominent neurologists. Potential underlying factors include infection, postoperative complications, anesthesia methods, and other intriguing factors. As an auxiliary examination, the implementation of apraclonidine is suggested. It is important to note that the downstream effects of sympathetic nerve lesions are not as sensitive as the upper ones. However, activation of the sympathetic nerves through apraclonidine (an alpha-adrenergic agonist) can alleviate ptosis and miosis by sensitizing these nerves (20). The development of artificial technology, particularly facial recognition, has shown limitless potential in assisting with the diagnosis of Horner’s syndrome. This technology automatically detects HS from facial images and has demonstrated promising results (21).

In cases of post-thyroidectomy HS, a conservative treatment approach is often preferred, involving neurotrophic drugs (22). In this particular case, it is recommended to prescribe Mecobalamin, along with vitamin B1, under the guidance of an experienced neurologist for neural repair. Remarkably, the effectiveness of this treatment can be validated based on the alleviation of symptoms, providing evidence for the availability of conservative treatment. However, it is important to acknowledge that long-term follow-up may show no improvement in some cases, leading to cosmetic disfigurement and potential mental disorders, which present significant challenges (23).

As a sporadic complication following endoscopic thyroid surgery, HS deserves significant attention. The surgical procedure holds a critical position and must be executed with utmost care, particularly regarding the preservation of anatomical structures, critical vessels, and nerves. A key factor in reducing the incidence of postoperative HS is the intact and precise separation of the prevertebral fascia and carotid sheath (24). As mentioned earlier, thermal damage caused by surgical instruments plays an essential role in the development of HS. Intermittent-intraoperative neural monitoring (I-IONM) effectively detects the distribution of important nerves around the thyroid gland, particularly facilitating the monitoring and protection of the recurrent laryngeal nerve from thermal damage (25, 26). Surgeons face the challenge of adapting to the limited vision of endoscopy, which lacks tactile feedback compared to conventional surgery. This poses a greater difficulty in preserving nerves during the procedure. It is recommended that surgeons carefully consider the merits and drawbacks of conventional and endoscopic surgery approaches to determine the optimal operative approach.

In summary, although the morbidity rate of postoperative HS is low, prompt diagnosis and therapy are crucial for improving therapeutic efficacy. Surgeons should be vigilant in their diagnosis and treatment to achieve the best possible outcomes.

## 4. Conclusion

HS after thyroidectomy is a postoperative complication that should not be overlooked, particularly following endoscopic-assisted thyroid surgery. While most patients with postoperative HS can experience recovery through nutritional and neurological treatment, there have been reports of persistent weakness in upper eyelid elevation and constriction of the pupil. Compared with open surgery, laparoscopic thyroidectomy offers several advantages. First, it involves smaller surgical incisions, resulting in improved cosmetic outcomes. In addition, laparoscopic surgery ensures the effectiveness and safety of the procedure. Women particularly favor it. However, it is important to note that laparoscopic surgery relies heavily on clear and complete exposure to the operative field and the assistance of thermodynamic machinery for cutting.

One limitation of laparoscopic thyroidectomy is the presence of visual blind spots within the laparoscope's field of view. The neurovascular tissue surrounding the thyroid gland is intricate, making it susceptible to accidental injury during surgery. While the superior laryngeal and recurrent laryngeal nerve require exposure protection during thyroid surgery, other nerve plexuses, such as the cervical sympathetic plexus highlighted in this article, also play a crucial role in the body. Iatrogenic injury to these nerves can lead to postoperative nerve damage and systemic complications. The most frequently encountered complications following thyroidectomy include temporary or permanent hypocalcemia and vocal cord paralysis (27). These complications arise from inadequate preservation of the parathyroid glands and recurrent laryngeal nerve during the surgical procedure. Other less common complications include visible neck hematoma, seroma formation, and Horner's syndrome (28). The development of Horner's syndrome can be attributed to factors such as compression from a hematoma, iatrogenic injury, or damage to the surrounding cervical plexus during probing of the carotid sheath, resulting in ocular sympathetic nerve injury.

To minimize the risk of tissue damage and optimize patient recovery, surgeons should thoroughly understand the histological anatomical structure. They should operate cautiously, ensuring complete liberation of the target tissue while protecting peripheral nerves and blood vessels before resection. This approach aims to minimize damage to surrounding tissues and promote postoperative recovery, ultimately alleviating the patient's pain and achieving the best cosmetic results.

## Data Availability

The raw data supporting the conclusions of this article will be made available by the authors, without undue reservation.
